# Small-scale field testing of alpha-cypermethrin water-dispersible granules in comparison with the recommended wettable powder formulation for indoor residual spraying against malaria vectors in Benin

**DOI:** 10.1186/s13071-018-3071-6

**Published:** 2018-09-12

**Authors:** Nicolas Moiroux, Armel Djènontin, Barnabas Zogo, Aziz Bouraima, Ibrahim Sidick, Olivier Pigeon, Cédric Pennetier

**Affiliations:** 1 Institut de Recherche en Sciences de la Santé (IRSS), Bobo-Dioulasso, Burkina Faso; 2 MIVEGEC, IRD, CNRS, University of Montpellier, Montpellier, France; 3 Centre de Recherche Entomologique de Cotonou (CREC), Cotonou, Bénin; 4 Département de Zoologie, Faculté des Sciences et Techniques, Université d’Abomey-Calavi, Calavi, Bénin; 5 Agriculture and Natural Environment Department (D3), Walloon Agricultural Research Centre (CRA-W), Gembloux, Belgium; 6 Institut Pierre Richet (IPR), Bouaké, Côte d’Ivoire

**Keywords:** Indoor residual spraying, Malaria, *Anopheles*, Vector control, Trial, Alpha-cypermethrin, Benin

## Abstract

**Background:**

Pyrethroids are the most common class of insecticide used worldwide for indoor residual spraying (IRS) against malaria vectors. Water-dispersible granules (WG) are a pyrethroid formulation to be applied after disintegration and dispersion in water with less risks of inhalation than using the usual wettable powder (WP) formulation. The objective of this small-scale field study was to evaluate efficacy and duration of insecticidal action of a new alpha-cypermethrin WG (250 g a.i./kg) against susceptible *Anopheles gambiae* in comparison with the WHO reference product (alpha-cypermethrin WP, 50 g a.i./kg) on the most common indoor surfaces in Benin.

**Methods:**

Both formulations were applied at two target-dose concentrations in houses made of mud and cement in the Tokoli village in southern Benin. We measured the applied dose of insecticide by chemical analysis of filter paper samples collected from the sprayed inner walls. We recorded *An. gambiae* mortality and knock-down rates every 15 days during 6 months using standard WHO bioassays.

**Results:**

The alpha-cypermethrin WG formulation did not last as long as the WP formulation on both surfaces. The difference is higher with the 30 mg/m^2^ concentration for which the WP formulation reached the 80% mortality threshold during 2 months on the mud-plastered walls (3 months on cement) whereas the WG formulation last only one month (2 months on cement).

**Conclusions:**

The new WG formulation has a shorter efficacy than the WHO recommended WP formulation. In this trial, both the WG and WP formulations had low durations of efficacy that would need at least two rounds of spray to cover the entire transmission season.

## Background

During the last decade, insecticide-treated nets (ITN) became the major malaria vector control tool implemented in Africa, complemented by indoor residual spraying (IRS) in some specific contexts. Indeed, these tools target different periods of the mosquito life-cycle (host-seeking behavior and resting behavior, respectively). In 2015, National Malaria Control Programmes (NMCP) reported that about 106 million people worldwide were protected by IRS. Pyrethroids were the class of insecticides the most used for IRS [1]. Over the 59 countries that have implemented IRS in 2014, 43 declared using pyrethroids alone or in combination with other classes of insecticides [2].

Pyrethroids insecticides are usually available in wettable powder (WP) formulations that present some disadvantages. First, the particles in suspensions made from wettable powders are large and visible residues may be left on sprayed surfaces. Moreover, there is a risk of inhalation during mixing, as the dry particles can become airborne. Alternatively, water-dispersible granules (WG) are a formulation consisting of granules to be applied by spraying after disintegration and dispersion in water. There is less risk of inhalation of airborne particles from water-dispersible granules than from wettable powders [3].

Alpha-cypermethrin is among the 12 insecticides recommended by the World Health Organization Pesticide Scheme (WHOPES) for IRS [4]. Alpha-cypermethrin has been tested and recommended by the World Health Organization (WHO) as a wettable powder (WP) and aqueous suspension concentrate at a dosage between 20 mg/m^2^ and 30 mg/m^2^ with expected residual activity between 4–6 months [5].

A WG formulation of alpha-cypermethrin was tested in India [6]. This formulation showed residual efficacy for 13–15 weeks for the 20 mg/m^2^ application rate and 13–16 weeks for the 30 mg/m^2^ rate not significantly different than the WHO recommended alpha-cypermethrin WP formulation [7]. WG formulations of other pyrethroids such as deltamethrin have also been tested but not in comparison with WP formulations. Residual efficacy varied from zero to less than 11 weeks on mud surfaces [8, 9] and from one to more than 41 weeks on concrete walls [8–10].

The objective of the present small-scale field study was to evaluate efficacy and duration of insecticidal action of a new alpha-cypermethrin WG (250 g a.i./kg) at two application rates (20 mg a.i./m^2^ and 30 mg a.i./m^2^) against susceptible *Anopheles gambiae* in comparison with similar dosages of the reference product (alpha-cypermethrin WP, 50 g a.i./kg) on the most common indoor surfaces in Benin.

## Methods

### Study area

The study was carried out in Tokoli (6°27'30"N, 2°10'16"E) in the Ouidah-Kpomasse-Tori health district in southern Benin. The climate is sub-equatorial with two dry seasons (August-September and December-March) and two rainy seasons (April-July and October-November). The average annual rainfall is around 1200 mm. The average monthly temperatures vary between 27–31 °C. Houses in southern Benin are of three types [11] that are all present in Tokoli: mud-plastered houses, houses made with white sand and cement, and houses made with red sand and cement.

### Insecticides tested

We tested the following formulations of α-cypermethrin: (i) α-cypermethrin WG: wettable granule formulation of alpha-cypermethrin (250 g a.i./kg) manufactured by Tagros Chemicals India Limited, Chennai, Jhaver Centre, Rajah Annamalai Bulding, IV Floor, 72, Marshalls Road, Egmore Chennai-600 008, India (Batch number AG-01/13, manufacturing date May 2013); and (ii) α-cypermethrin WP : WHOPES recommended alpha-cypermethrin Wettable Powder (50 g a.i./kg) manufactured by Tagros Chemicals India Limited (Batch number Lot-01, manufacturing date February 2013).

### Study design

As previous findings showed no difference in insecticide efficacy and persistence applied on red or white sands mixed with cement [11], we retained only two types of house (mud-plastered and cement) for this study. We included in the study 50 houses (25 mud-plastered and 25 with cement walls) of 112 houses in the village. In each batch of 25, houses were randomly allocated to one of the 5 following arms (i.e. 5 houses per arm): (i) α-cypermethrin WG at the 20 mg/m^2^ ± 25% target dose (WG20); (ii) α-cypermethrin WG at the 30 mg/m^2^ ± 25% target dose (WG30); (iii) α-cypermethrin WP at the 20 mg/m^2^ ± 25% target dose (WP20); (iv) α-cypermethrin WP at the 30 mg/m^2^ ± 25% target dose (WP30); and (v) control (not sprayed).

Houses were sprayed between the 7th and 16th March 2014 at the end of the long dry season. Only one room per house was sprayed. Insecticide was applied once, using a hand-operated compression sprayer (Hudson X-Pert, Chicago, USA) fitted with a 1.5 bar control flow valve on the lance pressure and equipped with ceramic 8002E nozzle. The four walls were treated. The spraying was done by two well trained technicians. They attended a 4-days training with a WHOPES mandated expert just before the beginning of spraying.

### Safety precautions

Safety precautions regarding mixing, handling and spraying the insecticide followed standard WHOPES procedures as outlined in [12]. Spray men used recommended/necessary protective clothing. They were given an information sheet in French and were briefed on possible adverse effects and the need to fully comply with safety instructions. Spray men were advised that in the event of any discomfort, they would be subjected to medical examination and care.

Rooms were sprayed after the personal effects of the householders have been removed and/or protected by craft paper. The windows, the floor, and the doors were also protected by craft paper during the spray. The householders were advised about safety precautions in order to avoid any risks during and after the spray. They were advised to remain out of the rooms during the spray and up to 3 h after spraying. They were told that it is required to protect them from coming in contact with fumes of the insecticide spray. The adult householders were advised to ask their children not to intentionally touch the sprayed walls for at least one day after spraying since the walls remained wet for about a day. After a room has been sprayed, it is essential that walls are not scrubbed or mutilated or plastered until the end of the study. The householders were therefore advised not to do so as part of the informed consent form. The householders were also advised that in the event of an adverse effect or illness due to fever, they should approach the Medical Officer at the closest health centre for treatment but may also seek advice/assistance from our institutions (IRD and CREC) at the contact details given in the consent form.

### Adverse effects on spray men and householders

Spray men were interviewed using a questionnaire at the end of a day of spraying, the following morning and one week after. Moreover, our team visited each household one week and one month after spraying to record adverse effect on the inhabitants using a questionnaire administered to the household heads.

### Residual activity

Standard WHO bioassays [13] were carried out on days 1, 15, 30, 45, 60, 90, 120, 150 and 180 after spraying, using laboratory-reared, susceptible females of *An. gambiae* (Kisumu strain). Mosquitoes were reared in insectary conditions (27 ± 3 °C, 60–80% relative humidity and a 12:12 h light and dark cycle). Ground cat food was used to feed larvae and 10% sucrose solution (with rabbit blood twice per week) to feed adult females. Batches of 10 unfed mosquitoes, 3–5 days old were put in a standard WHO cone and applied for 30 min (exposure time) on the four walls of each selected room. After 60 min, knock down (KD) mosquitoes were counted and all mosquitoes were kept for 24 h in the laboratory to assess mortality at 24 h.

### Chemical analysis

Before spraying, Whatman papers (10 × 10 cm) were attached to the four inner walls of each of the selected rooms before spraying, and collected 24 h post-spraying for chemical residue analysis. Positions of filter paper on the wall were marked to avoid carrying out cone bioassays at such spots. Each paper sample was packed in aluminum foil separately and put in labeled bags. The packed samples were stored in a fridge at 4 °C temperature before sending them to the WHO collaborating centre, Gembloux, Belgium for α-cypermethrin dosage.

### Statistical analysis

The α-cypermethrin content measured by chemical analysis on filter papers was compared between the 20 mg/m^2^ and 30 mg/m^2^ spraying objectives (for both formulations and both surfaces) and between the WP and WG formulations (for both spraying objectives and both surfaces) using *t*-tests (normality of the data and equality of variance were verified using D'Agostino-Pearson normality tests and *F*-tests, respectively).

Mortality and KD rates measured with WHO bioassays were analysed using binomial response mixed effect models with a random intercept for houses (to deal with possible auto-correlation among bioassay performed in a same room). Covariates used in the model as fixed effects were the treatment, the surface (mud or cement), the time after spraying (log-transformed) and interactions. The ‘glmer’ function of the *lme4* package [14] in the software R [15] was used for this analysis. The fitted models were used to predict (using the ‘predict’ function in R) the day when mortality fall under the efficacy threshold of 80%. Confidence intervals of predictions were computed using a code published on [16].

## Results

**Fig. 1:**
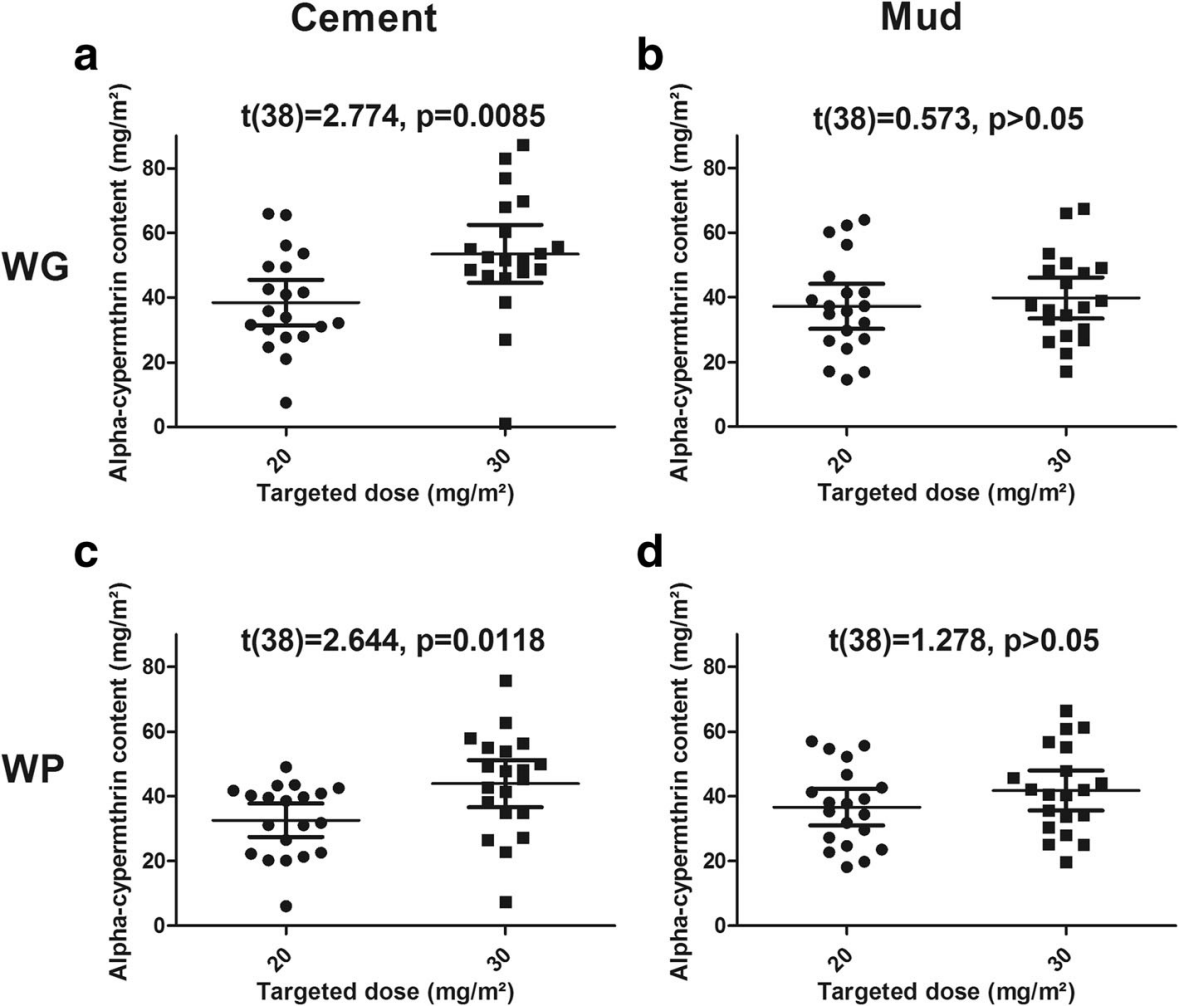
Comparison of applied and target doses (20 mg/m^2^ or 30 mg/m^2^) of insecticide according to the formulation (WP or WG) and the wall surface (mud or cement). Mean with 95% confidence interval are shown. *t*-test statistics and *P*-values are provided

On cement walls, chemical analysis of filter papers indicates that the mean concentrations of WP20 and WP30 were 32.60 mg/m^2^ (95% CI: 27.39–37.80) and 43.92 mg/m^2^ (95% CI: 36.62–51.21), respectively (Fig. 1a). On the same surface, the mean concentrations of WG20 and WG30 were 38.45 mg/m^2^ (95% CI: 31.48–45.41) and 53.47 mg/m^2^ (95% CI: 44.53–62.42), respectively (Fig. 1c). The differences in α-cypermethrin contents between the WP and WG formulations applied on cement walls were not significant (*t*_(38)_ = 1.408, *P* = 0.167 and *t*_(38)_ = 1.733, *P* = 0.091 for the 20 mg/m^2^ and 30 mg/m^2^ target doses, respectively). On mud walls, chemical analysis of filter papers indicates that the mean concentration of WP20 and WP30 were 36.62 mg/m^2^ (95% CI: 30.91–42.32) and 41.77 mg/m^2^ (95% CI: 35.55–47.99), respectively (Fig. 1b). On the same surface, the mean concentration of WG20 and WG30 were 37.23 mg/m^2^ (95% CI: 30.31–44.14) and 39.79 mg/m^2^ (95% CI: 33.47–46.11), respectively (Fig. 1d). The differences in α-cypermethrin contents between the WP and WG formulations applied on mud walls were not significant (*t*_(38)_ = 0.143, *P* = 0.886 and *t*_(38)_ = 0.467, *P* = 0.643 for the 20 mg/m^2^ and 30 mg/m^2^ target doses, respectively). The mean applied to target dose ratio was 1.64 (95% CI: 1.61–1.67), exceeding expectation. As expected, α-cypermethrin contents on cement wall were significantly higher with the 30 mg/m^2^ targeted doses (for both the WG and WP formulations) than with the 20 mg/m^2^ target dose (Fig. 1a, c). However, on mud walls, we were not able to find any difference between papers from rooms sprayed at 20 mg/m^2^ or 30 mg/m^2^ (Fig. 1b, d).

Over 40 filter papers coming from the 10 control houses, 39 showed a concentration of alpha-cypermethrin lower than the limit of detection (i.e. < 4 mg/m^2^) and one was just above (6.9 mg/m^2^).

**Fig. 2:**
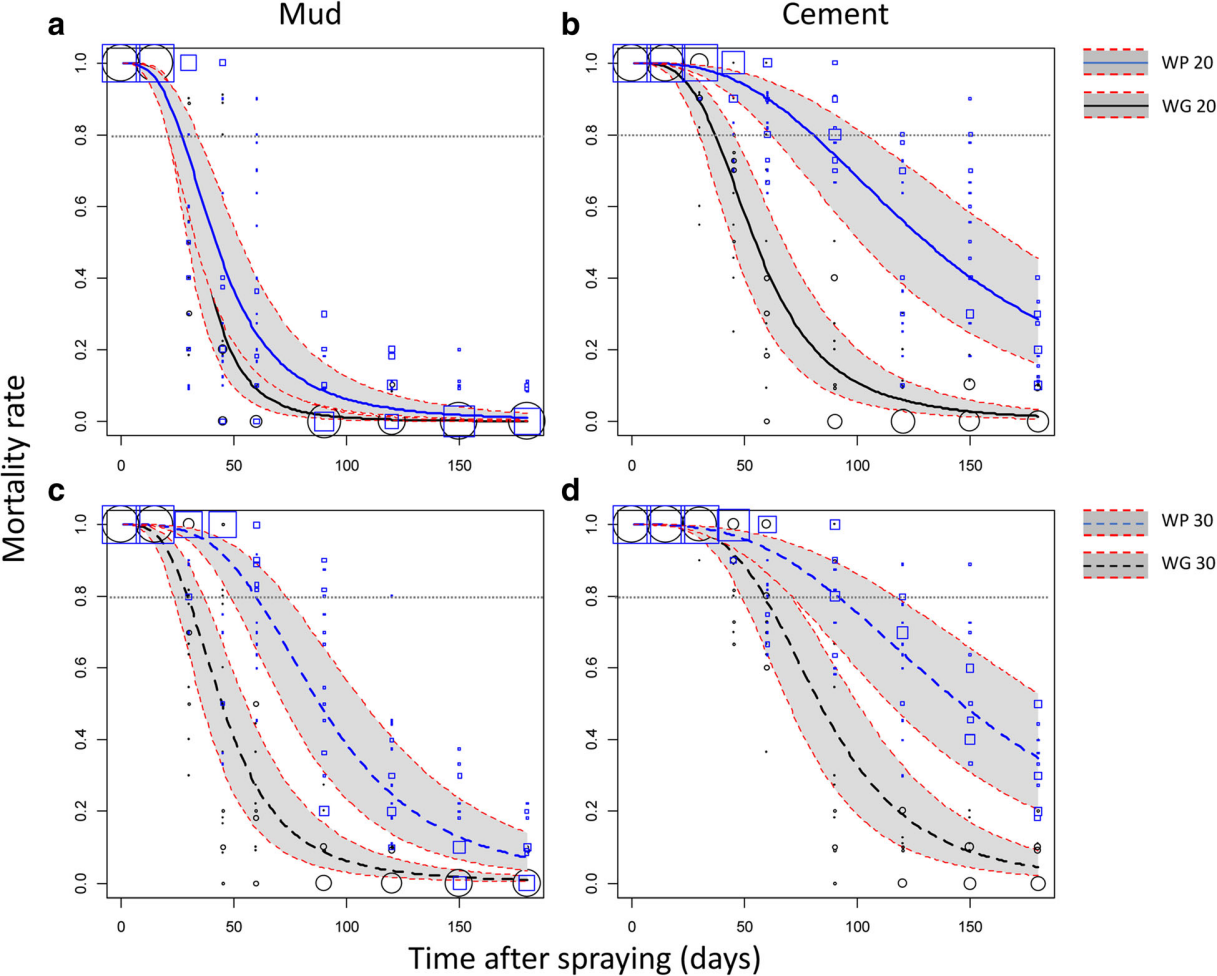
Efficacy (mortality) over time of indoor residual spraying of the WP and WG formulations of Alphacypermethrin against susceptible *An. gambiae*. Mortality rates were predicted from a binomial-response mixed effect model. Formulation WP (blue lines) and WG (black lines) at the 20 mg/m^2^ (**a**, **b**; solid lines) or 30 mg/m^2^ (**c**, **d**; dashed lines) targeted dose applied on mud (**a**, **c**) or cement walls (**b**, **d**) are compared. Grey areas are 95% confidence interval of predicted means. Mortality values measured on the field and used to fit the regression model are shown as blue squares (WP) and black circles (WG) of size proportional to the number of values (max = 20)

**Fig. 3:**
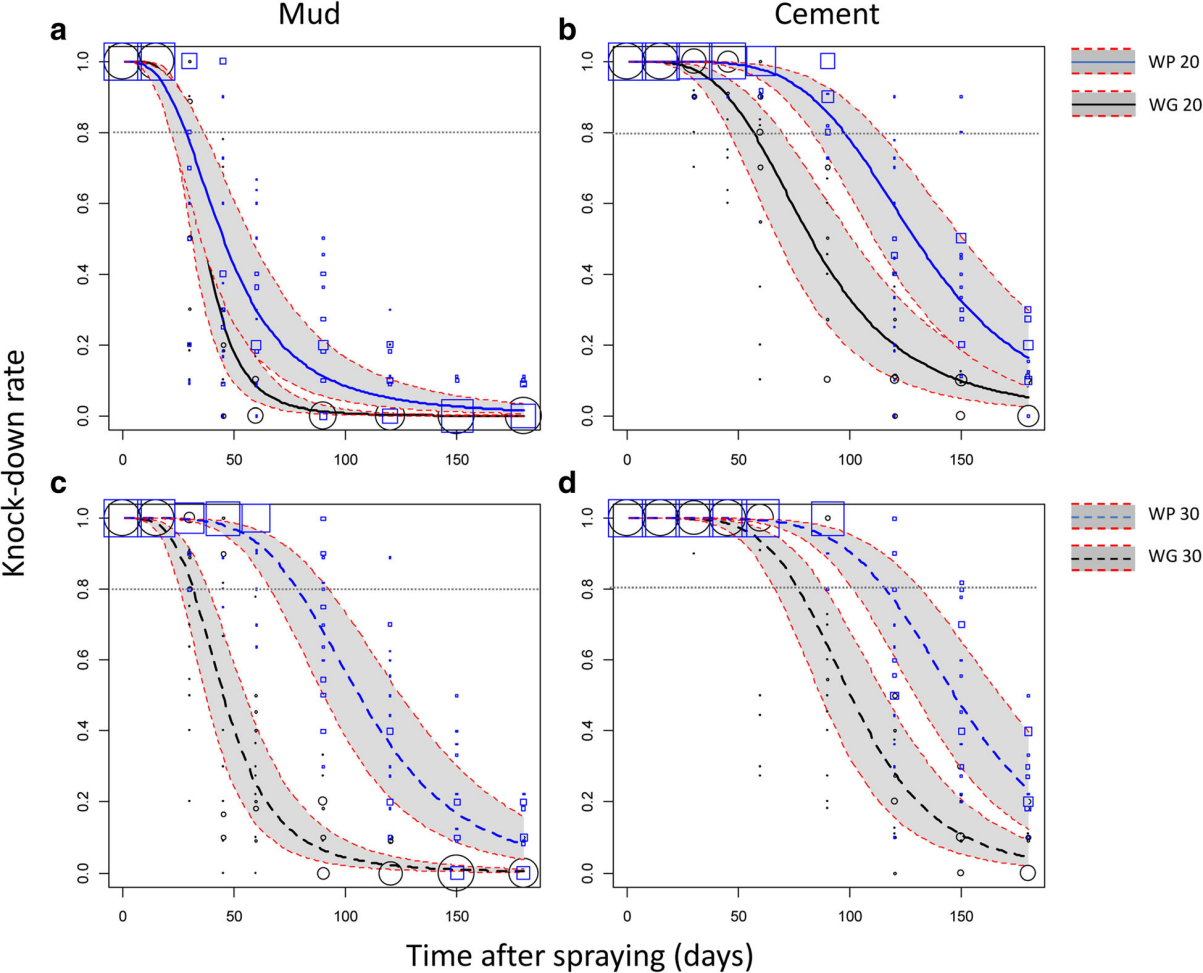
Efficacy (knock-down effect) over time of indoor residual spraying of the WP and WG formulations of Alphacypermethrin against susceptible *An. gambiae*. Knock-down (KD) rates were predicted from a binomial-response mixed effect model. Formulation WP (blue lines) and WG (black lines) at the 20 mg/m^2^ (**a**, **b**; solid lines) or 30 mg/m^2^ (**c**, **d**; dashed lines) targeted dose applied on mud (**a**, **c**) or cement walls (**b**, **d**) are compared. Grey areas are 95% confidence interval of predicted means. KD values measured on the field and used to fit the regression model are shown as blue squares (WP) and black circles (WG) of size proportional to the number of values (max = 20)

On the mud-plastered walls, the mortality model indicated that the WG20 and WP20 treatments efficacy failed significantly under the 80% threshold 26 and 27 days after spraying, respectively (Fig. 2a). The WG30 treatment was efficient until the 30th day after spraying. In contrast, the reference WP30 treatment was significantly more persistent with an induced mortality ≥ 80% until the 60st day (Fig. 2c). The same trends were observed for the KD rate (Fig. 3a, c) except for the WP30 formulation that maintained a KD rate ≥ 80% until the 79th day.

On the cement walls, mortalities induced by the WG formulation failed under the 80% threshold after 37 and 59 days when applied at the 20 mg/m^2^ and 30 mg/m^2^ target doses, respectively (Fig. 2b, d). In comparison, mortalities induced by the reference WP formulation failed under the 80% threshold after 81 and 92 days when applied at 20 mg/m^2^ and 30 mg/m^2^, respectively (Fig. 2b, d). Regarding the KD rate induced by the WG formulation, it failed under 80% after 57 and 77 days when applied at the 20 mg/m^2^ and 30 mg/m^2^, respectively (Fig. 3b, d). With the reference WP formulation, the KD rate failed under 80% after 98 and 117 days for the 20 mg/m^2^ and 30 mg/m^2^ target doses, respectively (Fig. 3c, d). In the control rooms, average mortality was 0.77 % (95% CI: 0. 5–1) 24 h post-exposure.

Two weeks after spraying, 6 household heads over 40 receiving an IRS treatment declared having experienced adverse effects (Table 1). They declared having experienced skin itching (*n* = 2), runny nose (*n* = 3), sneezing (*n* = 3), eye watering (*n* = 1), headache (*n* = 2) and nausea-vomiting-stomach pain (*n* = 1). One month after spraying, only two household heads having received the WG30 or WP30 treatments declared having experienced adverse effects [runny nose (*n* = 1), sneezing (*n* = 2), eye watering (*n* = 1)]. Spray men did not reported any adverse effect as well as householder of the control arm.

**Table 1:** Number of householders declaring adverse effects two weeks and one month after spraying according to the treatment arm

Time of survey	Treatment	No. of householders surveyed	Number of householders declaring adverse effects^a^						
			Skin itching	Nose running	Sneezing	Eye watering	Headache	Nausea, vomiting, stomach pain	At least one adverse effect
After 2 weeks	WG20	10	1	1	1	1	1	0	2
	WG30	10	0	1	2	0	0	0	2
	WP20	10	1	1	0	0	0	0	1
	WP30	10	0	0	0	0	1	1	1
	control	10	0	0	0	0	0	0	0
After 1 month	WG20	10	0	0	0	0	0	0	0
	WG30	10	0	1	1	0	1	0	1
	WP20	10	0	0	0	0	0	0	0
	WP30	10	0	0	1	0	0	0	1
	control	10	0	0	0	0	0	0	0

^a^Only adverse effects that were declared by at least one householder are listed. Facial burning, eye irritation, excessive sweating, experiencing bad smell after spraying, blurred vision, slurred speech, muscle twitching or other symptoms were not cited by any of the surveyed householders

## Discussion

In the present study, the residual efficacy of a new WG formulation of α-cypermethrin for IRS was tested on most common indoor surfaces (mud and cement) against a susceptible strain of *Anopheles gambiae* in Benin and was compared to the WHO recommended WP formulation.

This small-scale trial showed that the α-cypermethrin WG formulation efficacy did not last as long as the WP formulation on both surfaces. The difference is higher with the 30 mg/m^2^ concentration for which the WP formulation reached the 80% mortality threshold during two months, whereas the WG formulation last only one month on the mud-plastered walls. The same trend is observed for the cement surface on which the efficacy was ≥ 80% mortality during approximately three months for the WP formulation and less than two months for the WG formulation. This indicates that the new WG formulation was not as persistent as the reference WP formulation. These results contrasts with those of Uragayala et al. [6] in India who found that the WG formulation was as efficient as the reference WP formulation with durations of efficacy higher than three months whatever the target dose or the wall surface. Such differences observed between Benin and India might be due to the different *Anopheles* species used for bioassay, differences in wall surfaces and in climatic conditions (the Indian trial was performed during the dry season while the Beninese trial was almost entirely performed during the rainy season). Durations of efficacy of α-cypermethrin WP and deltamethrin WG formulations measured on mud or cement walls in other trials [8–10, 17, 18] were comprised between zero and 11 months. This interval, that includes our observations, illustrates the very large variability of IRS efficacy. Probable reasons for this variability have been cited above.

The short residual efficacy of both formulation in our trial indicated that multiple rounds of spraying should be needed to protect population for the entire malaria season that everywhere exceed four months in Benin. Moreover, it is notable that almost all α-cypermethrin contents measured on filter papers were above the targeted doses. If targeted doses had not been exceeded, we would have expected that durations of efficacy would have been shorter. Also, our experiment was made with susceptible strains of *An. gambiae*. In Benin, and almost everywhere in Africa, malaria vectors have developed resistances to pyrethroid insecticides [19–21]. Therefore, we would expect that residual efficacy of α-cypermethrin IRS against natural population of *An. gambiae* would be considerably shorter than shown in this study. Consequently, unless the epidemiological advantages of two rounds of spraying would be demonstrated, and because IRS are expensive to implement for resource-limited countries [22], such interventions should be limited to areas where the transmission season is short and where resistance to pyrethroid has not spread.

We found a difference in residual efficacy between the insecticides applied on mud and cement surfaces. This observation, already made in southern Benin [23], highlights the potential difference in residual efficacy of IRS between rural areas where the majority of houses is mud-plastered and the urban areas where cement surfaces are more common.

In tropical environments, mosquitoes that are KD have little chances to recover because of the biomass of potential predators and scavengers (e.g. ants, spies and geckos) [24]. The KD rate is therefore a highly relevant criterion for evaluation of IRS residual efficacy.

The chemical analysis showed a high variability of α-cypermethrin content within the filter papers attached on the inner walls. Despite the great experience of the sprayers, the training they attended just before the sprays and the supervision by a WHOPES mandated expert, insecticides concentrations applied on the walls were higher than expected. This illustrates the importance of providing, systematically, measured concentrations of insecticides when reporting results of field and semi-field evaluations of IRS. It is noteworthy that in operational conditions of IRS implementation, local operators may gain experience because of the scale of the operation. Nevertheless, independent evaluation of spraying quality should help improve operational procedures and go with Phase III evaluation studies [13].

However, the contents measured in houses that received the WP20 and WG20 treatment where still in the WHOPES recommended application dose of 20–30 mg/m^2^ ± 25% [7]. This makes the efficacy results obtained with the 20 mg/m^2^ target dose reliable since applied doses fall into the interval of the 30 mg/m^2^ WHOPES recommended application dose.

On cement wall, we find a positive relationship between the targeted dose and the concentrations measured on filter papers. It was expected to find the same on mud walls but we were not able to evidence such a relationship. However, we observed a higher mortality and longer efficacy on mud wall treated at the 30 mg/m^2^ with the WP formulation. This indicated that walls treated at 30 mg/m^2^ received more insecticide that those treated at 20 mg/m^2^ but the analysis of filter papers failed to detect it. As mud walls are more porous (and have therefore a higher sorption rate) than cement walls [25], we hypothesise that a significant but unknown proportion of the insecticide migrated from the filter papers into the mud wall. If confirmed, this issue might be easily solved by inserting an inert plastic sheet between the wall and the filter paper.

Adverse effects were reported only in houses having received an IRS treatment but sample sizes (i.e. 10 households/ arm) were too small to allow statistical comparisons between arms with a sufficient power. However, 15% (6/40) of surveyed householders declared having experienced adverse effects. This proportion is consistently higher than what was found with the same formulations in India [6].

## Conclusions

The tested α-cypermethrinWG formulation applied at a WHOPES recommended dose of 20–30 mg/m^2^ ± 25% (i.e. the WG20 and WP20 treatments) reached the cut-off point of 80% mortality during less than two months whatever the wall surface. This efficacy level was lesser than the WHO recommended α-cypermethrin WP formulation (almost three months before failing under 80% mortality) when applied on cement walls. When applied on mud-plastered walls, both formulations failed to exceed one month of efficacy. Because of these low durations of efficacy, we do not recommend the use of these formulations in Benin, where more than two rounds of spray should be needed to cover the entire transmission season.

## Abbreviations

a.i.: active ingredient
CI: confidence interval
CREC: Centre de Recherche en Entomologie de Cotonou
IRD: Institut de Recherche pour le Développement
IRS: indoor residual spraying
ITN: insecticide-treated net
KD: knock dawn
NMCP: National Malaria Control Program
WG: water-dispersible granules
WHO: World Health Organisation
WHOPES: World Health Organisation Pesticides Evaluation Schema
WP: wettable powder
